# Disruption of CCL2 in Mesenchymal Stem Cells as an Anti-Tumor Approach against Prostate Cancer

**DOI:** 10.3390/cancers15020441

**Published:** 2023-01-10

**Authors:** Quoc Thang Bui, Kuan-Der Lee, Yu-Ching Fan, Branwen S. Lewis, Lih-Wen Deng, Yuan-Chin Tsai

**Affiliations:** 1 International Ph.D. Program for Cell Therapy and Regeneration Medicine (IPCTRM), College of Medicine, Taipei Medical University, Taipei 110301, Taiwan; 2 Department of Medical Research, Taichung Veterans General Hospital, Taichung 407219, Taiwan; 3 Ph.D. Program for Cancer Molecular Biology and Drug Discovery, College of Medical Science and Technology, Taipei Medical University and Academia Sinica, Taipei 110301, Taiwan; 4 Graduate Institute of Cancer Biology and Drug Discovery, College of Medical Science and Technology, Taipei Medical University, Taipei 110301, Taiwan; 5 Department of Biochemistry, Yong Loo Lin School of Medicine, National University of Singapore, MD 7, 8 Medical Drive, Singapore 117596, Singapore; 6 NUS Center for Cancer Research, Yong Loo Lin School of Medicine, National University of Singapore, 14 Medical Drive, Singapore 117599, Singapore; 7 National University Cancer Institute, National University Health System, 5 Lower Kent Ridge Road, Singapore 119074, Singapore; 8 Ph.D. Program for Cancer Molecular Biology and Drug Discovery, College of Medical Science and Technology, Taipei Medical University, Taipei 110301, Taiwan

**Keywords:** CCL2, mesenchymal stem cells, tumor associated macrophages, tumor microenvironment, CD11b^+^Ly6G^−^

## Abstract

**Simple Summary:**

Cancer cells and mesenchymal stem cells (MSCs) secrete C-C motif chemokine ligand 2 (CCL2), a small protein that attracts tumor-associated macrophages (TAMs), which promotes malignant progression, such as drug resistance and metastasis. In addition, MSCs can also be recruited by CCL2; thus, a local milieu consisting of tumor cells, MSCs, and TAMs can promote tumor progression via a CCL2-dependent paracrine. The aim of the current study was to examine the functions of endogenous CCL2 of MSCs in cancer biology. Using a genetic engineering technique, we blocked the CCL2 expression in murine bone marrow-derived MSCs (CCL2 KO MSCs) and analyzed the effects on cancer progression. We found that CCL2 KO MSCs showed anti-tumor function when injected with murine prostate cancer cells into mice. The inhibitory effect was associated with an increase of CD45^+^CD11b^+^ mononuclear myeloid cells in tumors.

**Abstract:**

Background: MSCs are known to secrete abundant CCL2, which plays a crucial role in recruiting TAMs, promoting tumor progression. It is important to know whether disrupting MSC-derived CCL2 affects tumor growth. Methods: Murine bone marrow-derived MSCs were characterized by their surface markers and differentiation abilities. Proliferation and migration assays were performed in order to evaluate the functions of MSCs on cancer cells. CCL2 expression in MSCs was reduced by small interfering RNA (siRNA) or completely disrupted by CRISPR/Cas9 knockout (KO) approaches. An immune-competent syngeneic murine model of prostate cancer was applied in order to assess the role of tumor cell- and MSC-derived CCL2. The tumor microenvironment was analyzed to monitor the immune profile. Results: We confirmed that tumor cell-derived CCL2 was crucial for tumor growth and MSCs migration. CCL2 KO MSCs inhibited the migration of the monocyte/macrophage but not the proliferation of tumor cells in vitro. However, the mice co-injected with tumor cells and CCL2 KO MSCs exhibited anti-tumor effects when compared with those given tumor cell alone and with control MSCs, partly due to increased infiltration of CD45^+^CD11b^+^Ly6G^−^ mononuclear myeloid cells. Conclusions: Disruption of MSC-derived CCL2 enhances anti-tumor functions in an immune-competent syngeneic mouse model for prostate cancer.

## 1. Introduction

The androgen receptor (AR) plays a key role in the progression of prostate cancer, and androgen-deprivation therapy (ADT) is the first line of treatment against advanced metastatic prostate cancer after surgery of the primary site [1]. While ADT exhibits high efficacy against androgen-sensitive tumor cells at the beginning, patients eventually develop metastatic castration-resistant prostate cancer (CRPC) [2]. At this stage, many tumors have acquired mutations that overcome the castrated level of testosterone (e.g., amplification of AR gene or autogenic induction of androgen) [3,4]. Therefore, targeting AR function remains the mainstay of therapeutic approaches to CRPC [5].

Many molecular mechanisms underlying castration resistance or androgen independence have been identified in CRPC cells [6]; however, other factors in the complex tumor microenvironment (TME) also contribute to malignant progression. TAMs are capable of switching the functions of some AR antagonists from suppression to activation [7], and M2-polarized macrophages are associated with immunosuppression and poor survival rate [8]. Although several chemokines are capable of recruiting TAMs to the TME [9], the chemoattractant C-C motif ligand 2 (CCL2), alias MCP1, is the key factor and is known to be induced in prostate cancer in response to AR inhibition [10,11]. In addition to TAMs, MSCs can promote CRPC progression and metastasis by converting to cancer-associated fibroblasts [12,13]. Importantly, MSCs also secrete CCL2 [14,15]. MSC-derived CCL2 contributes to cancer progression not only by attracting TAMs but also by facilitating their M2-polarization [16]. Since tumor-derived CCL2 also recruits MSCs to the TME [17], an established CCL2 paracrine among tumor cells, MSCs, and TAMs in TME can support malignant progression.

Increased serum CCL2 has been demonstrated as a promising biomarker in diagnosis and prognosis [18,19,20]. Targeting CCL2 using its neutralizing antibody has been successful in mouse xenograft models in preventing prostate cancer metastasis [21,22]; however, it was reported that accelerated relapse and metastasis may occur once the antibody administration is interrupted [23]. Since CCL2-neutralizing antibodies inhibit not only TAMs but also all the normal monocyte/macrophages in the body (e.g., bone marrow), which contributes to systemic side effects, we thought to utilize the tumor-homing ability of MSCs [13] and to develop a genetically-engineered cellular therapy. We confirmed that tumor cell-derived CCL2 was crucial in regulating the migration activities of MSCs and in tumor growth using a syngeneic mouse prostate cancer model. The expression of endogenous CCL2 in MSCs was transiently inhibited by small interfering RNA (siRNA) or permanently by CRISPR/Cas9 KO. The role of CCL2-suppressed MSCs in cancer growth was examined by proliferation assays in vitro and a syngeneic mouse tumor model.

## 2. Materials and Methods

### 2.1. Cells and Conditioned Media Preparation

Mouse prostate cancer cell lines (TRAMP-C1, TRAMP-C2 and TRAMP-C3), mouse macrophages (RAW 264.7), and human monocytes (THP-1) were obtained from the American Type Culture Collection (ATCC, Manassas, VA, USA). Cells were maintained in the suggested culture medium according to the ATCC. Commercial mouse bone marrow-derived mesenchymal stem cells (BM/MSCs) were purchased from Cyagen (Santa Clara, CA, USA), and the cells were maintained following the manufacturer’s guidelines. The preparation of primary BM/MSCs was modified from an established procedure [24]. In brief, the bone marrow cells of 8-week-old C57BL/6 mice were flushed out with Dulbecco’s Modified Eagle’s Medium supplemented with 100 U/mL penicillin/streptomycin and 10% fetal bovine serum (GIBCO, Grand Island, NY, USA). By washing with phosphate buffer saline (PBS) and filtering with 70 μm nylon mesh filter (Falcon Cell Strainers, Thermo Fisher Scientific, Waltham, MA, USA), cells were cultured in a 5% CO_2_ incubator. Unbound cells were removed every 24 h for three consecutive days to enrich MSCs utilizing their plastic adherence property. The MSCs were further enriched from the flow through cells using an EasySep™ Mouse Mesenchymal Stem/Progenitor Cell Enrichment Kit (STEMCELL technologies, Vancouver, BC, Canada). An established procedure was modified in order to isolate MSCs in the TME [25]. In brief, MSCs in the TRAMP-C1-derived tumor-infiltrating leukocytes were isolated by the same Mouse Mesenchymal Stem/Progenitor Cell Enrichment Kit (STEMCELL Technologies). Culture media from the MSCs (ca. 80% confluence) were collected as conditioned media and stored at −80 °C.

Similar to the isolation of MSCs in the TME, an established procedure was modified in order to prepare the CD11b-positive (CD11b^+^) cells [25]. In brief, CD11b^+^ TRAMP-C1-derived tumor-infiltrating leukocytes were enriched by magnetic beads using the EasySep™ mouse CD11b-positive selection kit II (STEMCELL Technologies).

### 2.2. In Vitro Cell Proliferation Analysis

Colony assay was performed with 500 cells seeded into each well in 12-well plates which were incubated with either the conditioned media or complete media. After seven days of incubation, cells were fixed and stained with 0.2% crystal violet. The images of colonies were taken and analyzed by Image J using the ColonyArea plugin [26].

A colorimetric cell viability kit was used to determine cell proliferation in vitro. In brief, the cells were seeded in 96-well plates for 24 h followed by treatment with either the conditioned media, mouse IL-28α, or IL-28β (ProSpec, Rehovot, Israel) for 1–3 days. A CCK-8 solution (Sigma-Aldrich) was applied to each well and incubated for 1 h. The measurement was done by reading the absorbance at 450 nm on an Epoch Microplate Spectrophotometer (BioTek Instruments, Winooski, VT, USA).

### 2.3. Western Blot Analysis

Cell lysates were processed with 6× Laemmli sample buffer, followed by sodium dodecyl sulfate-polyacrylamide gel electrophoresis (SDS-PAGE). Samples were transferred onto the polyvinylidene difluoride (PVDF) membranes and blocked with 5% milk in TBST (Sigma-Aldrich, St. Louis, MO, USA). Western blotting was performed with specific antibodies at 4 °C overnight: anti-CD11b antibody (ABclonal, Woburn, MA, USA) and anti-GAPDH antibody (GeneTex, Irvne, CA, USA) (Supplementary Table S1). Membranes were washed with TBST twice and incubated with an HRP-labeled antibody at room temperature for 1 h. After incubation with an HRP substrate (Western Bright ECL HRP Substrate, Advansta, San Jose, CA, USA), images were taken with an Amersham™ Imager 600 (GE Healthcare, Chicago, IL, USA).

### 2.4. Enzyme-Linked Immunosorbent Assay (ELISA)

Mouse CCL2 (Quantikine ELISA Kit; R&D Systems, Minneapolis, MN, USA), and mouse Interleukin 1 Receptor Antagonist (IL1RA) ELISA Kit (MyBioSource, CA, USA) were used to detect specific cytokine secretion. The procedure was performed following the manufacturer’s protocol. In brief, conditioned media (C.M.) were collected and stored at −80 °C until being measured. After incubation for 2 h at room temperature in 96-well ELISA plates, each well was washed and incubated with 100 µL of conjugate solution for 2 h. After washing, each well was incubated with 100 µL of substrate solution for 30 min, followed by the addition of 100 µL of stop solution. Measurements were made by reading the absorbance at 450 nm on an Epoch Microplate Spectrophotometer (BioTek Instruments, Winooski, VT, USA).

In order to analyze the cytokine/chemokine profile, the conditioned media of MSCs were collected and analyzed using a Proteome Profiler™ Array kit (R&D Systems) following the manufacturer’s guidelines.

### 2.5. Differentiation of MSCs and Staining Assays

Osteoblast differentiation was assayed with 10,000 cells/cm² in a basal culture medium. At 80% cell confluence, cultured media were changed to an osteogenic induction medium consisting of DMEM (GIBCO, Grand Island, NY, USA) containing 10% FBS, 100 U/mL of penicillin, 100 mg/mL of streptomycin, 50 μg/mL of vitamin C, 10 nM dexamethasone, and 10 mM β-glycerol-phosphate (Sigma-Aldrich). The media were changed every 3–4 days during the culture. Cells were induced to osteoblast differentiation for 28 days. After fixing, cells were stained with 40mM Alizarin red S (Sigma-Aldrich), pH 4.2 at room temperature (RT) for 30 min, followed by washing with water. The images of calcium acceleration were taken and analyzed.

Adipocyte differentiation was assayed with 10,000 cells/cm² in a basal culture medium. At 80% cell confluence, cultured media were changed to an adipogenic induction medium consisting of DMEM supplemented with 10% FBS, 100 U/mL of penicillin, 100 mg/mL of streptomycin, 1 μM dexamethasone, 500 μM 1-methyl-3-isobutyl-xanthine (IBMX), 1 μM rosiglitazone, 100 μM indomethacin, and 5 μg/mL insulin (Sigma-Aldrich). The media were changed every 3–4 days during the culture. Cells were induced to adipocyte differentiation for 21 days. After fixing, cells were stained with 0.5% Oil red O solution (Sigma-Aldrich) for 60 min at RT, followed by washing with water. The images of lipid droplets were taken and analyzed.

### 2.6. Flow Cytometry Analysis

Monitoring of bone marrow-derived MSCs was performed by a Mouse Mesenchymal Stem Cell Multi-Color Flow Kit (R&D Systems, Minneapolis, MN, USA). Briefly, cells were stained with specific antibodies (anti CD29-PE, anti-Sca-1-APC, anti-CD45-PerCP) or isotype controls (anti-IgG2A-APC, anti-IgG2A-PE, anti-IgG2A-PerCP) in the dark at 4 °C for 45 min.

In order to investigate the profile of the tumor microenvironment, the tumors were isolated into single cells, blocked with FcR Blocking Reagent (Miltenyi Biotec, Gaithersburg, MD, USA), followed by staining with fluorescent dye–conjugated anti-CD45, anti-CD11b, and anti-Ly6G (Supplementary Table S2) in the dark at 4 °C for 30 min. The profiles were analyzed by Attune NxT Cytofluorimeter (Invitrogen, Carlsbad, CA, USA).

### 2.7. Generation of CCL2-KO Cell Lines and Conditioned Media Preparation

Guide (g)RNA targeting mouse CCL2 exons 1 (NM_011333.3) was generated based on the CRISPR/CRISPR-associated protein 9 (Cas9) editing technique. Mouse CCL2 gRNA sequences were selected from a CRISPR-designed website (https://chopchop.cbu.uib.no, accessed on 16 October 2018) and cloned into pSpCas9(BB)-2A-Puro (PX459) V2.0, a gift from Feng Zhang (Addgene plasmid # 62988) (Supplementary Table S3). Following transient transfection, MSCs or TRAMP-C2 cells were treated with puromycin (2.5 µg/mL) to select puromycin-resistant clones. CCL2-KO clones were confirmed by several approaches: ELISA for CCL2 secretion, DNA sequencing for mutation profiles, and a cytokine/chemokine analysis (Proteome Profiler Mouse Cytokine Array, R&D Systems). Genomic DNA isolated from KO clones was amplified by a polymerase chain reaction (PCR) using specific primers (Supplementary Table S4) to identify mutation sequence.

### 2.8. siRNA Knockdown of the il1rn & ccl2 Expression

MSCs were seeded into six-well plates at a density of 2 × 10^⁵^ cells/well. When the cell reached ~80% confluence, small interfering RNA targeting CCL2 (Supplementary Table S5) or IL1RN (Sigma-Aldrich) was transfected into MSCs using RNAiMAX (Invitrogen). After 20 h, the medium was replaced with complete media. After two days of cultivation, the supernatants were collected and stored at −80 °C until use.

### 2.9. Migration Assay

Migration assays were performed in a Transwell Chamber with 8 μm pore size filters (BD Biosciences, Corning, NY, USA). A total of 2.5 × 10^⁴^ cells were seeded in 1% FBS medium into the upper chamber and allowed to migrate/invade toward the bottom section containing either the conditioned or complete media for 24 h. Migrated cells were fixed, stained with 0.2% Crystal violet, and counted in 2–3 randomly chosen fields. For each condition, two replicate chambers were assayed. For THP-1, 2.5 × 10⁴ cells were seeded into the upper chamber with 1% FBS and allowed to migrate through the bottom section for 6 h. The average number of migrated cells per field was calculated for each group.

### 2.10. Syngeneic Prostate Cancer Mouse Model

An animal experiment was performed in accordance with a protocol approved by the Taipei Medical University Animal Care and Use Committee (approval no.: LAC-2017-0274, Taipei, Taiwan). For tumor growth analysis, 10^⁶^ TRAMP-C2 cells with or without 10^⁶^ MSCs cells were subcutaneously injected into 8-week-old male C57BL/6 mice (NLAC, Taipei, Taiwan). Tumor sizes were quantified according to the formula: length (mm) × width (mm)^2^/2, and mice were sacrificed to collect tumors at size ~900 (mm³).

### 2.11. In Vitro Cytotoxicity Assay

In vitro cytotoxicity assay was performed with a cytotoxicity assay kit (CFSE, 7AAD) (Abcam, Cambridge, UK). Briefly, TRAMP-C2 was stained with CSFE at RT for 20 min before co-culturing with parental or CCL2 KO MSCs with a tumor-to-MSC ratio from 3:1 to 1:1. After two days of incubation, the staining dye 7AAD was added to stain all necrotic cells red by binding to the DNA of membrane-compromised cells. A positive control (dead cells) was performed by incubating the cells at 56 °C for 15 min, followed by staining with 7-AAD. The percentage of dead TRAMP-C2 cells (CSFE^+^ 7AAD^+^ population) was analyzed by Attune NxT Cytofluorimeter (Invitrogen, Carlsbad CA, USA).

### 2.12. Statistical Analysis

All data were presented as the mean ± standard deviation (SD). Differences between groups were analyzed by Student’s *t*-test. Statistical calculations were performed with Prism analytical tools (GraphPad Software). A *p*-value of < 0.05 was considered significant.

## 3. Results

### 3.1. Inhibition of Tumor Growth by CCL2 Knockout (KO) in a Syngeneic Prostate Cancer Model

**Figure 1:**
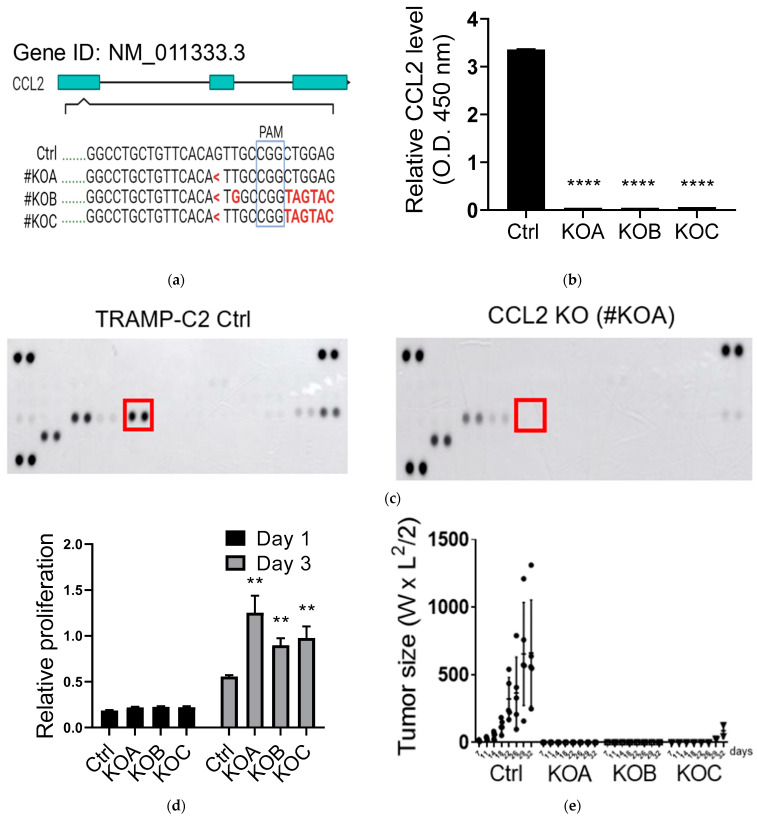
The requirement of tumor-derived CCL2 in tumor growth. (**a**) Establishment of CCL2 knockout (KO) in TRAMP-C2 cell line (Ctrl vs. KOA-KOC). PAM: the protospacer-adjacent motif. <: deletion; Red: mutation. (**b**) Comparison of CCL2 protein secretion in cell media (Ctrl vs. KOA-KOC) by ELISA. (**c**) Comparison of cytokine/chemokine profiles of TRAMP-C2 (Ctrl) and its CCL2 KO derivative (KOA); Red box: CCL2. (**d**) Comparison of cell proliferation rates in vitro among TRAMP-C2-derived clones (Ctrl vs. KOA-KOC); n = 6. (**e**) Comparison of tumor growth rates in vivo. Immune competent syngeneic mice (C57BL/6) were injected with different TRAMP-C2-derived clones (Ctrl/n = 5 vs. KOA-KOC/n = 3 each). Student’s *t*-test. ** *p* < 0.01. **** *p* < 0.0001.

In order to study the role of CCL2 in a network consisting of tumor cells, MSCs, and TAMs in the TME, we disrupted the CCL2 gene in the transgenic adenocarcinoma of the mouse prostate (TRAMP)-C2 cell line [27]. We isolated three CCL2 knockout (KO) clones (KOA-KOC, Figure 1a) and confirmed the absence of CCL2 (Figure 1b). We also compared the cytokines/chemokines secreted into the conditioned media and confirmed again the loss of CCL2 expression (red box, Figure 1c). When we monitored the in vitro proliferation, all of the CCL2 KO clones showed increased rates compared to the control (Figure 1d). Many studies have shown the positive regulatory role of CCL2 in tumor growth; indeed, we also observed reduced proliferation in TRAMP-C1 following CCL2 knockdown by siRNA (Supplementary Figure S1). Therefore, the selected CCL2 KO clones may already bypass the CCL2-CCR2 signaling and even acquire mutations involved in mitogenic pathway, leading to enhanced proliferation in vitro. However, all of the KO clones failed to form tumors when subcutaneously injected into immune competent, syngeneic mice (Figure 1e). It was shown that mice deficient in CCL2 exhibited inhibitory effects to skin carcinogenesis [28], showing its role in maintaining the TME. Our results confirmed that tumor cell-derived CCL2 plays an essential role in the syngeneic prostate cancer model, possibly due to the recruitment of many immunosuppressive cells (e.g., TAMs).

### 3.2. Tumor Cell-Derived CCL2 Is Crucial in Recruiting Mesenchymal Stem Cells (MSCs)

**Figure 2:**
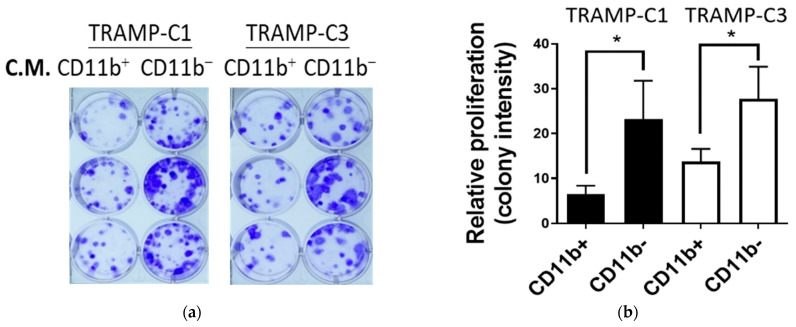

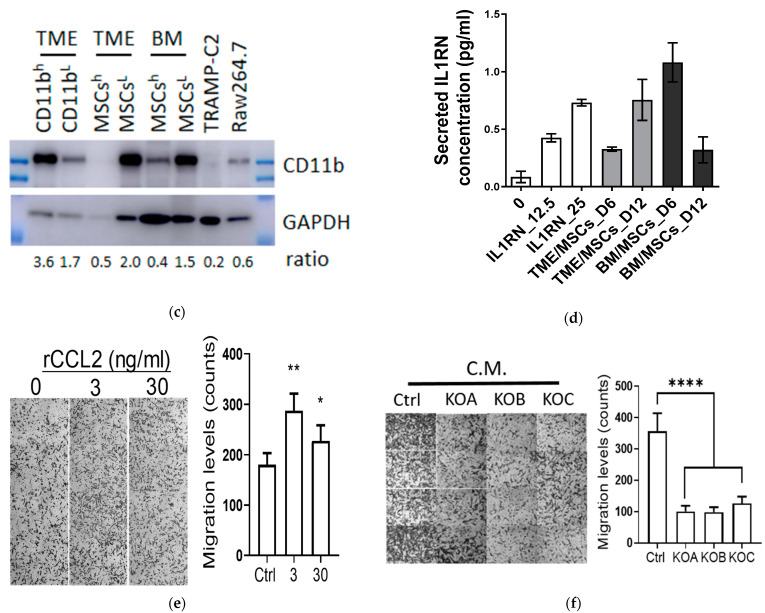
Tumor cell-derived CCL2 is involved in regulation of the migration activities of MSCs (**a**) Colony-formation assays using conditioned media (C.M.) collected from the CD11b^+^ and CD11b^−^ leukocytes in the TRAMP-C1-derived tumors. C.M. was applied to two mouse prostate cancer cells (TRAMP-C1 and TRAMP-C3). (**b**) Colony numbers were quantified and compared (right). n = 3. (**c**) Comparison of the CD11b expression of different cell populations by Western blotting assay. Single cell suspension collected from the TME or bone marrow (BM) was treated with either a CD11b or MSCs enrichment kit. CD11b^h^ and MSC^h^: enriched populations. CD11b^L^ and MSC^L^: non-enriched populations. Ratio: images were quantified and presented as CD11b/GAPDH. (**d**) Detection of IL1RN secretion in the conditioned media of MSC^h^ populations from the TME (TME/MSCs) and bone marrow (BM/MSCs). D6 and D12: condition media collected on day 6 (D6) and day 12 (D12). (**e**) Transwell analyses of BM/MSCs in response to different concentrations of recombinant CCL2 (rCCL2); n = 4. (**f**) Transwell analyses of BM/MSCs in response to different C.M. collected from TRAMP-C2-derived CCL2 KO clones (Ctrl vs. KOA–KOC); n = 4. Student’s *t*-test. * *p* < 0.05. ** *p* < 0.01. **** *p* < 0.0001.

By analyzing the cytokine/chemokine profiles of the tumor cell-derived TME using mouse Lewis lung cancer and transgenic adenocarcinoma of the mouse prostate cell lines, we demonstrated that a population deficient in cluster differentiation 11b (CD11b) contributed to the anti-inflammatory environment in the TME, partly via secretion of a natural anti-inflammatory cytokine, IL1RN, and promoted tumor cell proliferation [29]. Indeed, we observed again the promotional effect of the CD11b^−^ population in the TME (Figure 2a,b). It was shown that MSCs were deficient in CD11b [30]; therefore, we analyzed the CD11b expression in primary MSCs derived from either the TME or bone marrow (BM) (Figure 2c). We confirmed that the ratio between CD11b and GAPDH drastically decreased in MSC-enriched populations (MSCs^h^ vs MSCs^L^) both in the TME (0.5 vs. 2.0) and BM (0.4 vs. 1.5) (Figure 2c; Supplementary Figure S2). It was shown that the MSC-derived IL1RN plays an important role [31,32]. By monitoring the MSCs-derived C.M. collected on different days (D6, D12), we confirmed the secretion of IL1RN by MSCs (Figure 2d). In summary, our results suggest the recruitment of MSCs in the TME.

We hypothesized that the CCL2 paracrine in the TME establishes a network consisting of tumor cells, MSCs, and TAMs; consistently, it was suggested that breast cancer-derived CCL2 is involved in the homing effect of human MSCs [17]. Thus, we examined the role of tumor-derived CCL2 in regulating the migration activities of the MSCs. In order to secure the abundance and consistency of MSCs for further investigation, we used commercially available mouse bone marrow-derived MSCs and confirmed again their low expression of CD11b (Supplementary Figure S3). Consistently, using recombinant CCL2 protein enhanced the migration activities of MSCs (Figure 2e). Furthermore, the conditioned media collected from all the CCL2 KO TRAMP-C2 cell lines (KOA-KOC) showed significantly reduced migration activities of MSCs (Figure 2f). In summary, our results suggest that tumor cell-derived CCL2 is involved in the recruitment of MSCs to TME.

### 3.3. Inhibitory Effects of Condition Media Collected from the Bone Marrow-Enriched Mesenchymal Stem Cells (MSCs) with CCL2 Knockdown by Specific Small Interfering RNA (siRNA)

**Figure 3:**
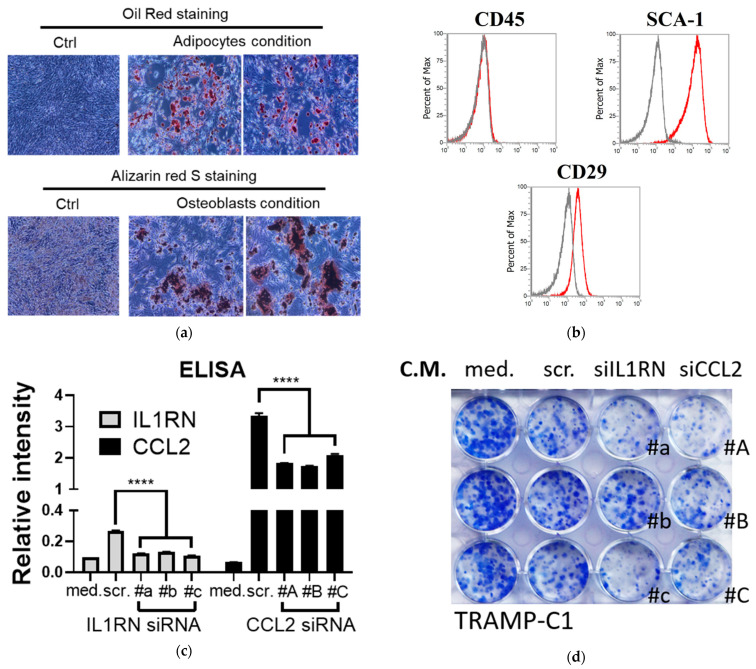

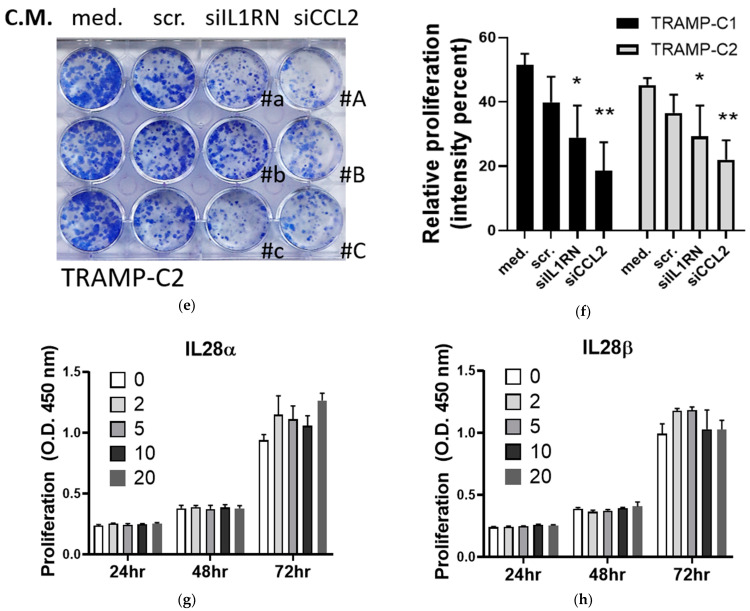
Anti-proliferation function of the conditioned media (C.M.) derived from the MSCs with knockdown of IL1RN and CCL2. (**a**) Differentiation of MSCs to adipocytes (oil red) and osteoblasts (Alizarin red S). MSCs without differentiation treatment serve as control (Ctrl). (**b**) Characterization of MSC markers using flow cytometry to detect several surface markers. Black: isotype antibody control. Red: specific antibody. (**c**) Confirmation of knockdown efficiency by measuring IL1RN and CCL2 protein secretion in the MSCs treated with respective siRNAs: siIL1RN (#a–#c) and siCCL2 (#A–#C). Protein secretion was measured by ELISA. (Ctrl vs. KOA-KOC) by ELISA; n = 3; med: fresh medium, scr: siRNA control. (**d**,**e**) Colony-formation assays using C.M. collected from the siRNA-treated MSCs in panel (**a**). C.M. was applied to TRAMP-C1 [panel (**d**)] and TRAMP-C2 [panel (**e**)]. (**f**) Colony numbers and staining intensities were quantified and compared; n = 3. (**g**,**h**) Proliferation analysis using TRAMP-C2 cell line in response to IL-28α [panel (**g**)] and IL-28β [panel (**h**)] recombinant proteins (ng/mL); n = 4. Student’s *t*-test. * *p* < 0.05. ** *p* < 0.01. **** *p* < 0.0001.

Although MSCs exhibit a tumor promotion function [12,13], it was shown that MSCs also exerted anti-proliferation effects on bone metastatic prostate cancer via an IL-28-mediated signaling pathway and contributed to the development of drug-resistant clones [33]. Thus, we asked whether endogenous IL1RN and CCL2 expressed in MSCs were involved in growth regulation. We utilized the MSCs from bone marrow and confirmed their properties in differentiation (Figure 3a) and surface marker expression (CD45^−^Sca-1^+^CD29^+^, Figure 3b). After confirmation of the source of MSCs, the expression of endogenous IL1RN and CCL2 was reduced by the siRNA approach (Figure 3c), and the C.M. was collected for further analysis. We performed a colony assay in order to monitor the effects of C.M. in both TRAMP-C1 (Figure 3d) and TRAMP-C2 (Figure 3e). While we did not observe a significant reduction of proliferation using the C.M. from parental MSCs (med. vs. scr., Figure 3d,e), downregulation of IL1RN (siIL1RN) and CCL2 (siCCL2) showed enhanced anti-proliferation effects on both cell lines (Figure 3f). However, the inhibitory effects were not dependent on IL-28 as previously reported [33], since the cell line was not sensitive to either IL-28α or IL-28β recombinant protein (Figure 3g,h). In summary, our results suggest that disrupting MSC-derived IL1RN and CCL2 can enhance anti-proliferation effects.

### 3.4. Establishment of CCL2 Knockout (KO) in Mesenchymal Stem Cells (MSCs)

**Figure 4:**
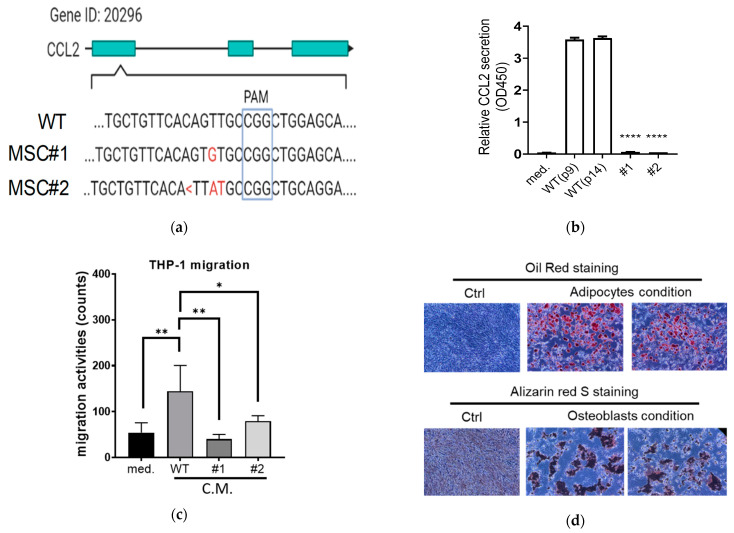

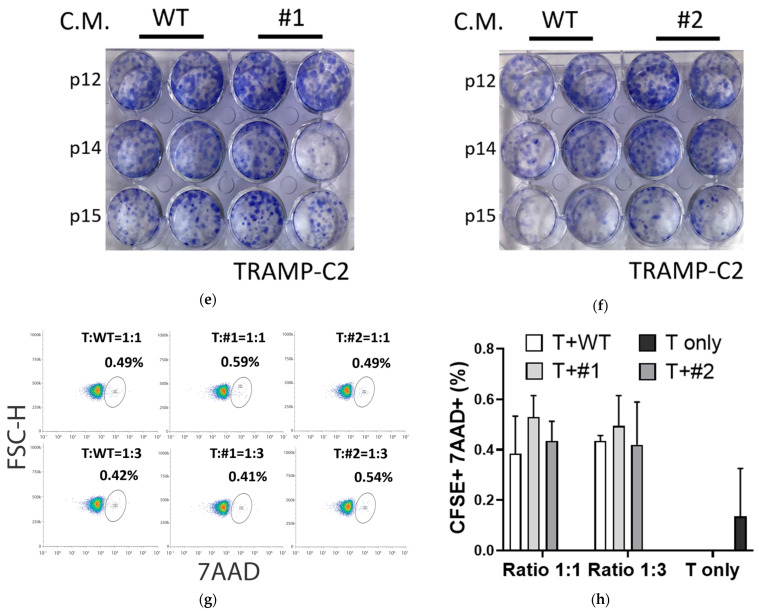
Lack of anti-proliferation function of MSCs in vitro. (**a**) Establishment of CCL2 knockout (KO) in bone marrow-derived MSCs. (WT vs. MSC#1-#2). PAM: the protospacer adjacent motif. <: deletion. Red: mutation. (**b**) Comparison of CCL2 protein secretion in cell media by ELISA. Different passage numbers of MSCs (p9 and p14) were compared with CCL2-KO MSCs (#1 and #2). med: negative control of the fresh medium. (**c**) Transwell analyses of THP-1 cells in response to different conditioned media (C.M.) collected from MSCs (WT vs. #1-#2). n = 3. (**d**) Differentiation of CCL2-KO MSCs to adipocytes (oil red) and osteoblasts (Alizarin red S). MSCs without differentiation treatment serve as control (Ctrl). (**e**,**f**) Colony-formation assays using C.M. collected from the MSCs [panel (**e**): WT vs. #1; panel (**f**): WT vs. #2]. C.M. collected from different passage numbers (p12, p14, p15) was applied to TRAMP-C2; n = 2. (**g**) Co-culture assay using TRAMP-C2 cells (T) labeled with CFSE and then mixed with different MSCs (WT, #1, #2). After 48 h, the mixture was stained with 7AAD, and the CFSE^+^7AAD^+^ population was analyzed by flow cytometry. (**h**) Quantification of CFSE^+^7AAD^+^ population in TRAMP-C2 (T) collected from panel (**g**); n = 3. Student’s *t*-test. * *p* < 0.05. ** *p* < 0.01. **** *p* < 0.0001.

Many studies have aimed to apply MSCs as a therapeutic approach in clinics [34]; we hypothesized that genetically engineered MSCs with CCL2 KO can enhance the anti-proliferation activities against tumors. To test this idea, we utilized CRISPR/Cas9 system to disrupt the CCL2 gene in mouse bone marrow-enriched MSCs (#1-#2, Figure 4a). We confirmed the mutations of these KO clones (#1-#2, Figure 4a) and their lack of CCL2 expression by ELISA (Figure 4b). In addition, we collected the C.M. and tested their abilities to recruit monocytes using the THP-1 cell line. As shown in Figure 4c, the transwell activities were lost using the C.M. collected from the CCL2 KO clones. When we performed a differentiation assay, CCL2 KO in the MSCs did not affect their abilities to differentiate into either adipocytes or osteoblasts (Figure 4d).

Since the C.M. from CCL2 siRNA-treated MSCs showed enhanced anti-proliferation effects (Figure 3f), we further examined the functions of the CCL2 KO clones in proliferation. However, compared with parental MSCs (WT), we did not observe inhibitory effects using C.M. collected from several passages of the CCL2 KO clones (Figure 4e,f). This discrepancy may be due to adaption (e.g., crosstalk from other signaling pathways) or acquired mutations, replacing the need of CCL2 for CCR2 signaling in the CCL2 KO MSCs. Thus, we asked whether MSCs may trigger cytotoxic effects in tumor cells via direct cell-to-cell interaction. In order to address this issue, we performed a co-culture assay using TRAMP-C2 and the MSCs. The TRAMP-C2 cell line was labeled with a protein-conjugating dye (carboxyfluorescein diacetate succinimidyl ester, CFSE) and then mixed with MSCs. After incubation for 2 days, the percentage of dead cells in the CFSE^+^ population was measured by using a DNA intercalating dye (7-aminoactinomycin D, 7AAD) (Figure 4g). Although we tested two different tumor-to-MSC ratios (1:1 and 1:3), the CFSE^+^ 7AAD^+^ percentages were very low and not statistically different from TRAMP-C2 (T) only in all the experimental settings (Figure 4h). In summary, our results show that the CCL2 KO MSCs do not induce anti-proliferation effects against TRAMP-C2 cells in vitro.

### 3.5. Enhanced Anti-Tumor Effects of the CCL2 KO MSCs in a Syngeneic Prostate Cancer Model

**Figure 5:**
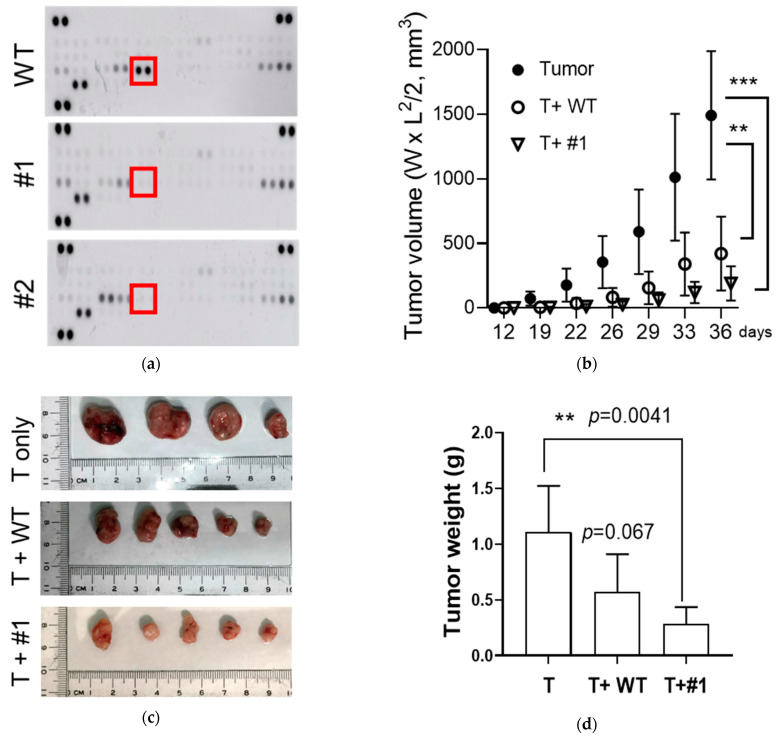

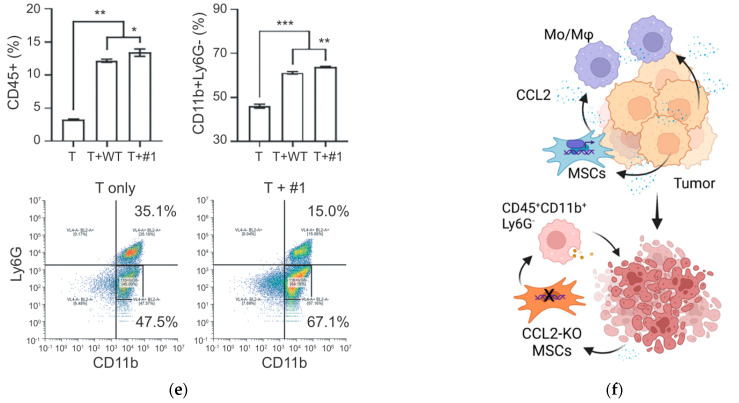
Enhanced anti-tumor effects of the CCL2 knockout (KO) MSCs in vivo. (**a**) Monitoring cytokine/chemokine profiles in C.M. collected from the MSCs (WT) and CCL2-KO MSCs (#1–#2). Red box: CCL2. (**b**–**d**) Comparison of tumor growth rates in vivo. Immune competent, syngeneic mice (C57BL/6) were co-injected with TRAMP-C2 (T) and MSCs (WT vs. #1). Time course of tumor size [panel (**b**)]. Images of the collected tumors from each group [panel (**c**)] and final tumor weights [panel (**d**)]. (**e**) Immune profile analysis of CD11b^+^LyG6^−^ populations in the tumor microenvironment. n = 3. (**f**) A working model. Prostate cancer and MSCs secret CCL2 to recruit monocyte/macrophage (Mo/Mφ). Using CCL2-KO MSCs to suppress tumor growth indireclty via CD45^+^CD11b^+^Ly6G^−^ mediated functions. Student’s *t*-test. * *p* < 0.05. ** *p* < 0.01. *** *p* < 0.001.

MSC-derived CCL2 is involved in the tumor-promoting functions of macrophages [16], and targeting CCL2 has been a therapeutic approach [21,22]. Although we did not observe the anti-proliferation effects of the CCL2 KO MSCs in vitro, we sought to examine their roles in the TME developed in an immune-competent syngeneic mouse prostate cancer model. We analyzed the profiles of secreted cytokine and confirmed again the lack of CCL2 in CCL2 KO MSCs clones (#1-#2, Figure 5a). Using a TRAMP-C2 cell line (TRAMP-C2/Luc-eGFP), expressing a fusion protein containing both an enhanced green fluorescent protein (EGFP) and luciferase (Luc), we found that the tumors co-injected with both CCL2 KO MSCs showed reduced growth rates compared with parental MSCs (T+WT vs. T+ #1/or #2, Supplementary Figure S4). However, mice injected with TRAMP-C2/Luc-eGFP cells only, without MSCs, did not show tumor formation either (tumor only, Supplementary Figure S4), which could be due to the anti-tumor immunogenic effects of the neo-antigens derived from both the EGFP and Luc genes. Therefore, we performed the animal study again using a parental TRAMP-C2 cell line without EGFP and Luc expression.

Surprisingly, compared with mice injected with parental TRAMP-C2 cells only, co-injection with either WT or CCL2 KO MSCs significantly reduced the tumor growth rates; however, the CCL2 KO (#1) exhibited enhanced inhibitory effects (Figure 5b). After we collected the tumors and measured their weights, only the tumor co-injected with CCL2 KO MSCs showed a reduction with statistical significance (T vs. T+#1, Figure 5c,d). Therefore, these results support that CCL2 KO MSCs exhibited enhanced anti-tumor functions. The distinct effects between in vitro (Figure 4) and in vivo (Figure 5) suggest that the MSCs established an anti-tumor microenvironment in the syngeneic tumor model. In order to address this issue, we analyzed the immune profile in the tumors and found a significant increase (~20%) of CD45^+^CD11b^+^Ly6G^−^ populations (Figure 5e). Since CD45^+^CD11b^+^Ly6G^−^ populations primarily contain monocytes and macrophages, our results suggest that the CCL2 KO MSCs promote anti-tumor responses via mononuclear myeloid cell-mediated pathways in the TME.

## 4. Discussion

The role of MSCs in prostate cancer remains unclear. While the malignant progression of prostate cancer was shown to be promoted by MSCs [12,13], it was also reported that MSCs can suppress growth via IL-28 mediated apoptosis [33]. Using C.M. collected from MSCs treated with siRNA targeting either IL1RN or CCL2, we also observed the inhibition of proliferation; however, the mechanism was not likely due to the IL-28 related pathway, since the tumor cells were not sensitive to exogenous IL-28 at high concentrations (Figure 3). Surprisingly, we did not observe a significant anti-proliferation effect when either the parental or CCL2 KO MSCs were co-cultured with tumor cells in vitro (Figure 4). It is possible that tumor cells are capable of compromising the anti-tumor functions of MSCs either by secreted cytokines or direct interactions; therefore, under the in vitro experimental setting as the co-culture assay, we only focused on the signaling between tumor cells and MSCs, recapitulating part of the communications in the tumor microenvironment. On the contrary, when the MSCs were co-injected with tumor cells in the immune competent syngeneic mice, the CCL2 KO MSCs indeed showed increased anti-tumor effects compared to wildtype MSCs (Figure 5d). Since MSCs do not express the CD45 marker, marked increase of CD45^+^ and CD45^+^CD11b^+^Ly6G^−^ mononuclear populations in the tumor microenvironment confirmed the immune modulating functions of MSCs.

Although both WT and CCL2 KO MSCs reduced tumor growth in mice injected with the parental TRAMP-C2, we suspected that the WT MSCs can be tumor-promoting in certain situations. In mice injected with the TRAMP-C2/Luc-eGFP cells, only co-injection of WT MSCs (T+WT) showed significant tumor growth, compared with those with CCL2 KO MSCs (T+#1 and T+#2) or no MSCs (Tumor only) (Supplementary Figure S4). Therefore, the anti-tumor mechanism against the TRAMP-C2/Luc-eGFP is different from that against the parental TRAMP-C2 in a syngeneic model. The anti-tumor immunity against TRAMP-C2/Luc-eGFP cells in the TME may be induced by the neo-antigens derived from Luc and EGFP foreign proteins, and only the WT MSCs can suppress this immunogenic environment leading to tumor growth. On the other hand, the parental TRAMP-C2 cell line is a selected clone from the TRAMP model [27], the anti-tumor effect observed in both WT and CCL2 KO MSCs was less likely to be due to immunogenic neo-antigens but rather to the elevated CD45^+^CD11b^+^Ly6G^−^ mononuclear population (Figure 5d). Our earlier studies demonstrated tumor type-specific TME in syngeneic mice in that parental TRAMP-C1 cells established an anti-inflammatory TME while a lung cancer cell line resulted in an inflammatory one [25,29]. Similar to TRAMP-C1, the parental TRAMP-C2 may exhibit an anti-inflammatory TME that influences the functions of the MSCs (WT and CCL2 KO), supporting mononuclear population and anti-tumor effects. Since the CCL2 KO MSCs showed enhanced anti-tumor effects compared to WT MSCs in mice injected with either TRAMP-C2 or its derived TRAMP-C2/Luc-eGFP cells, we proposed a working model depicting the potential therapeutic benefit of this genetically engineered cell therapy (Figure 5f). Further investigation is required to address whether tumor cells exhibiting inflammatory TME exhibit distinct immune responses to WT and CCL2 MSCs.

Neutrophils (CD45^+^CD11b^+^Ly6G^+^) occupy a high percentage of leukocytes in the TME; however, using the Ly6G marker, we showed that co-injection with MSCs reduced the neutrophil-to-mononuclear ratio in CD11b^+^ myeloid cells. Thus, although neutrophils can exhibit anti-tumor function, our results suggested that the mononuclear CD45^+^CD11b^+^Ly6G^−^ cells play an important role. The role of CD11b^+^ myeloid cells in tumor biology is controversial. It was reported that using CD11b neutralizing antibodies can sensitize the therapeutic effect of radiation by reducing infiltration of myeloid cells [35]; however, using a CD11b-activating molecule was shown to suppress tumor growth via pro-inflammatory macrophages, likely M1-polarized macrophages [36]. Consistently, a negative association between CD11b signaling and M2-polarized macrophages was shown in that CD11b deletion in macrophages induced anti-inflammatory cytokines, and immune suppressive signals inhibited CD11b expression [36]. Since MSCs were shown to increase CD11b expression in dendritic cells [37], it is possible that MSCs can maintain and activate CD11b signaling in the CD45^+^CD11b^+^Ly6G^−^myeloid cells, promoting their anti-inflammatory functions. Furthermore, MSC-derived CCL2 and CXCL12 form a complex to induce M2-polarized macrophages, and disrupting CCL2 evidently obstructs this process [16]. Thus, delivering CCL2 KO MSCs into the TME may prevent immunosuppressing TAMs and facilitate the M1-polarized macrophages, promoting anti-tumor function. However, it is not clear how the mice co-injected with both tumor cells and MSCs exhibited a drastic increase (~20%) in CD45^+^CD11b^+^Ly6^−^ population (Figure 5e). It is possible that abundant MSCs in the TME perturb the balance of different chemokines; consequently, it provides a unique niche favoring the recruitment or proliferation of CD45^+^CD11b^+^Ly6^−^ population. It would be interesting to study the underlying mechanism in the future.

In addition to CCL2, the C.M. collected from MSCs with the downregulation of IL1RN by the siRNA approach exhibited anti-proliferation activity against the prostate cancer cells (Figure 3). Although we do not know the effect in vivo on the TME, it was reported that MSC-derived IL1RN promoted differentiation of M2 macrophages, similar to the role of CCL2 [32,38]. Additionally, IL1RN is known to be the key anti-inflammatory factor of both human and murine M2 macrophages, suppressing the IL-1-related pro-inflammatory functions [39]. Based on our previous studies, using the TRAMP-C1-derived tumors collected from immune-competent mice indeed exhibited an anti-inflammatory TME partly due to IL1RN induction [25]. The source of IL1RN in the TRAMP-C1-derived tumors was likely from both the MSCs and M2-macrophages, but not tumor cells per se [29]. Based on these results, it would be interesting to know whether delivering IL1RN KO MSCs also enhances anti-tumor effects. Further investigation is required to address this issue.

## 5. Conclusions

Genetically engineered MSCs with CCL2 knockout can enhance the anti-tumor effect in an immune-competent syngeneic mouse model of prostate cancer.

## Data Availability

Data is contained within the article or supplementary material.

## Supplementary Materials

The following supporting information can be downloaded at: https://www.mdpi.com/article/10.3390/cancers15020441/s1, Figure S1: Monitor CD11b expression. Figure S2: CD11b expression in MSCs. Figure S3: tumor growth rate using TRAMP-C2 expressing a luciferase-eGFP fusion protein. Figure S4: Comparison of tumor growth rates using TRAMP-C2/Luc-eGFP cell line. Table S1: Antibodies for Western blotting; Table S2: Antibodies for Flow cytometry; Table S3: Guide RNA (gRNA) sequences targeting the CCL2 genes; Table S4: Primers for sequencing CCL2 KO; Table S5: siRNA sequences targeting the CCL2.
