# Supplementary material for: Disruption of CCL2 in Mesenchymal Stem Cells as an Anti-Tumor Approach against Prostate Cancer
Source: Cancers (Basel). 2023 Jan 10;15(2):441. doi: 10.3390/cancers15020441 (PMC9857005; doi:10.3390/cancers15020441)
Supplement: Supplementary file 1 [file cancers-15-00441-s001.zip › cancers-2117323-supplementary.pdf]

Supplementary Material

Disruption of CCL2 in mesenchymal stem cells as an anti-tumor approach against prostate cancer

Supplementary Figure S1

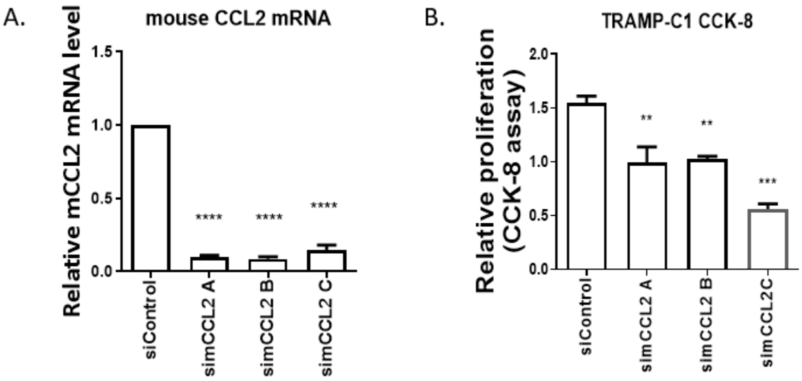

Monitoring effect of CCL2 siRNA in proliferation. A. A comparison of knockdown efficiencies of different siRNAs (simCCL2A-C). siControl serves negative control. B. Monitoring proliferation in TRAMP-C1 cell using CCK-8 assay.

Supplementary Figure S2

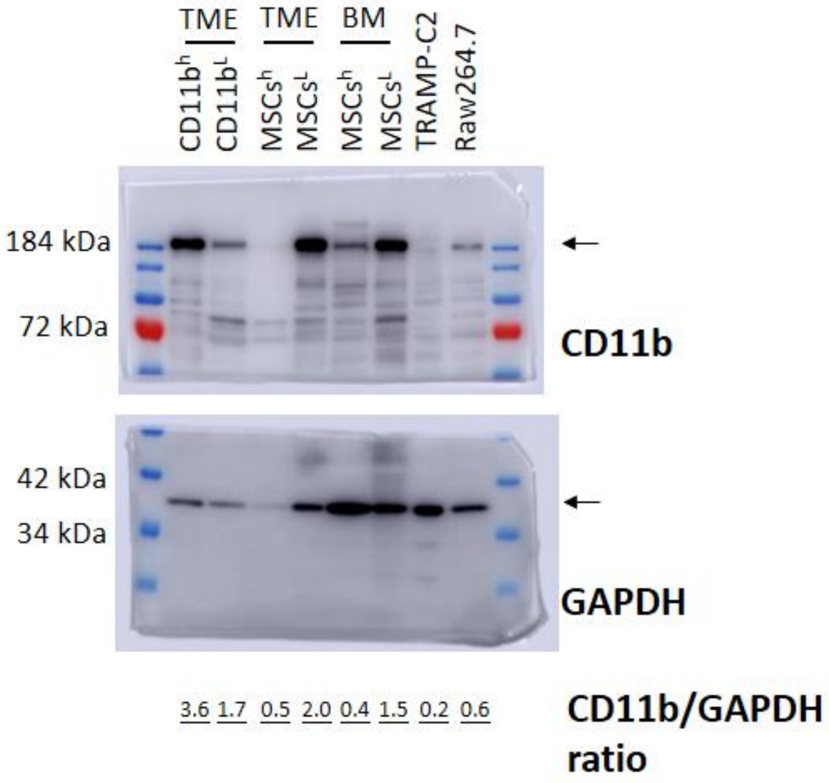

Monitor CD11b expression. Full images of the Western Blotting assay of Figure 2c. A comparison for different cell populations, leukocytes in the TME, bone marrow, and other cell lines. CD11b^h:

enriched populations of tumor-infiltrating leukocytes in the TRAMP-C1-derived tumors were isolated by a CD11b enrichment kit. MSC^{h}: enriched MSCs from the bone marrow (BM) by an MSCs enrichment kit. CD11b^{L} and MSC^{L}: non-enriched populations.

Supplementary Figure S3

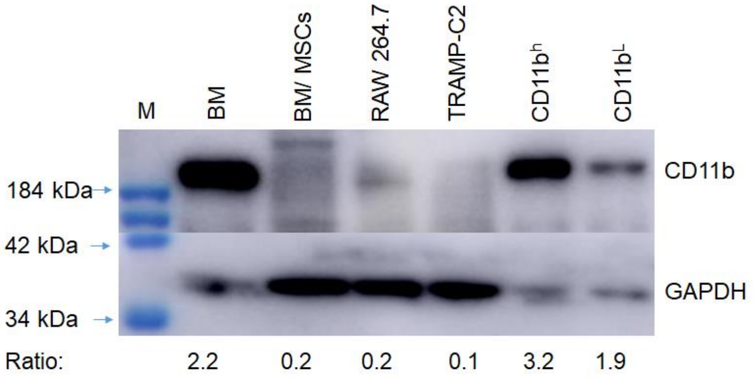

CD11b expression in MSCs. Comparison of CD11b expression in commercially purchased bone marrow-derived mouse MSCs (BM/MSCs) with other cell populations. CD11b^{h} and CD11b^{l} served as a positive and negative control, respectively.

Supplementary Figure S4

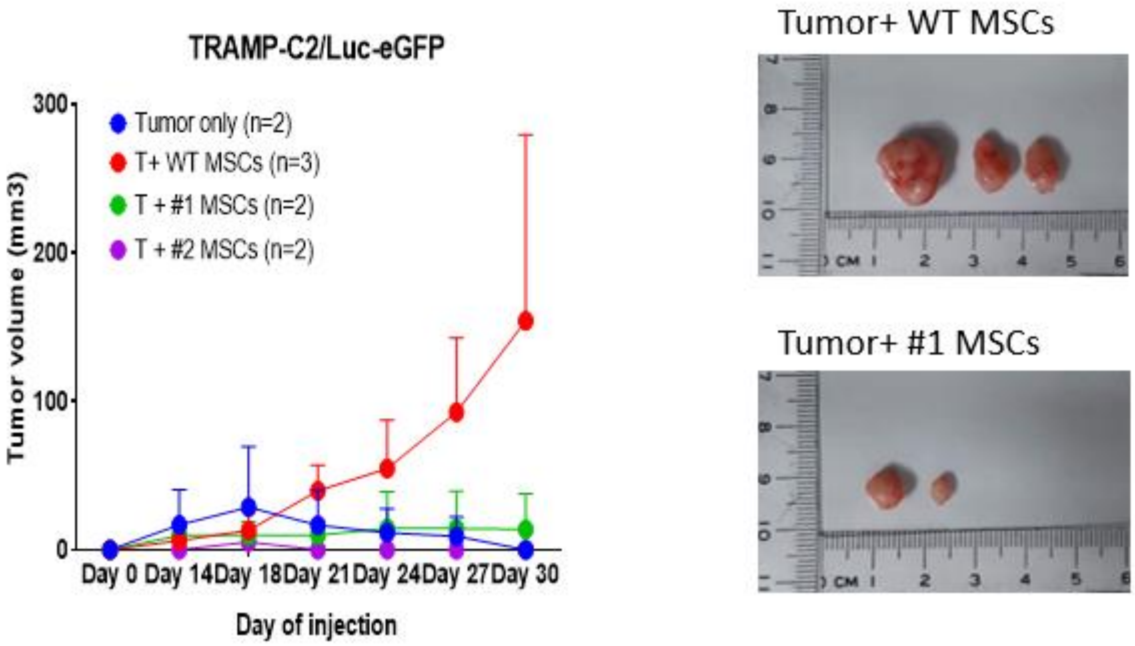

Comparison of tumor growth rates using TRAMP-C2/ Luc-eGFP cell line in a syngeneic mouse model. The TRAMP-C2 cell line stably expressing both EGFP and luciferase (Luc) genes (TRAMP-C2/Luc-eGFP) was established earlier. Immune competent, syngeneic mice (C57BL/6) were co-injected with TRAMP-C2/Luc-eGFP cells (T) and different MSCs (WT, KO #1, and KO #2). Tumor sizes were recorded at different days. WT: the parental BM/MSCs expressing CCL2. #1 & #2: the

two clones selected from the CCL2 KO MSCs. After sacrifice, only mice injected with tumor cells plus MSCs (WT & #1) had tumor formation. Mice injected with tumor cells only or with #2 MSCs did not show tumor formation.

Supplementary Table S1. Antibody information for Western blotting.

Primary antibody	Clonality	Source	Dilution	Secondary antibody	Source	Dilution
GAPDH	Polyclonal	GeneTex (GTX100118)	1/10000	anti-rabbit IgG	Croyez Bioscience	1/10000
CD11b	Polyclonal	ABclonal (A1581)	1/1000	anti-rabbit IgG	Croyez Bioscience	1/10000

Supplementary Table S2. Antibody information for Flow cytometry.

Primary antibody	Clonality	Source	Dilution
CD45	Monoclonal	BD Biosciences (560501)	1/100
CD11b	Monoclonal	BD Biosciences (563168)	1/50
Ly6G	Monoclonal	BioLegends (127607)	1/50

Supplementary Table S3: Guide RNA (gRNA) sequences targeting the CCL2 genes.

Gene	5'-3'
CCL2 F	GGCCTGCTGTTCACAGTTGC
CCL2 R	GCAACTGTGAACAGCAGGCC

Supplementary Table S4. Primer sequences for sequencing the INDEL mutation on KO genes.

Gene	5'-3'
CCL2 F	GCAGAGAGCCAGACGGGAG
CCL2 R	ACCTGACCATGTGAGACTATGCTATC

Supplementary Table S5. Small interfering RNA (siRNA) sequences targeting the CCL2 genes

Gene	5'-3'
CCL2 set A	GAUGUUAUGGUAUUCCCUU[dT][dT]
CCL2 set B	CUAAUGCAUCCACUACCUU[dT][dT]
CCL2 set C	CUCAAGCACUUCUGUAGGA[dT][dT]
